# Effects of Temperature and Humidity on the Fitness of Aphid Parasitoid, *Binodoxys communis*

**DOI:** 10.3390/insects16030264

**Published:** 2025-03-03

**Authors:** Shike Xia, Ningwei Ma, Peiling Wang, Yanhui Lu

**Affiliations:** 1 State Key Laboratory for Biology of Plant Diseases and Insect Pests, Institute of Plant Protection, Chinese Academy of Agricultural Sciences, Beijing 100193, China; xiashikee@163.com; 2 Key Laboratory for Xinjiang Oasis Agricultural Pest Management and Plant Protection Resources Utilization, Agricultural College of Shihezi University, Shihezi 832003, China; 15999299650@163.com (N.M.); wangpl69@126.com (P.W.); 3 Western Agricultural Research Center, Chinese Academy of Agricultural Sciences, Changji 831100, China

**Keywords:** *Binodoxys communis*, *Aphis gossypii*, temperature, humidity, parasitism efficiency, population expansion, biological control

## Abstract

*Binodoxys communis* is a dominant endoparasitoid of aphids in cotton fields, yet empirical evidence on how temperature and humidity regulate its growth, development, and reproduction remains limited. To address this gap, we assessed the effects of both constant and fluctuating temperature, as well as various combinations of temperature and humidity, on the longevity, parasitism, and fecundity of this parasitoid. Our results revealed that adult longevity of *B. communis* was longer at 20 °C and 25 °C while significantly shortened at a high temperature (35 °C). Similarly, the parasitism rate, female ratio, emergence duration, and offspring longevity of the parasitoid were all superior at 20 °C and 25 °C compared to 15 °C and 35 °C. Moreover, the longevity of both male (6.96 ± 0.10 d) and female (6.88 ± 0.07 d) parasitoids was significantly extended at 25 °C and 60% RH. Temperature had a marked impact on the parasitic capability of parasitoids, with the number of *Aphis gossypii* parasitized daily by *B. communis* being significantly higher at 25 °C than at 15 °C and 35 °C. Nevertheless, humidity and the interaction between humidity and temperature had no significant influence on parasitic capacity. The parasitism of *B. communis* followed the Holling-II model, with the highest daily maximum parasitism observed at 25 °C. In conclusion, our study showed that 25 °C positively enhanced the fitness of *B. communis*, providing a valuable reference for indoor population expansion and field release of *B. communis*, potentially enhancing its effectiveness as a biological control agent against aphids.

## Simple Summary

The growth and development of parasitoids are influenced by various abiotic factors, such as temperature and humidity. Our study examined the effects of constant and fluctuating temperatures and humidity on the fitness of *Binodoxys communis* indoors. The results indicated that at 20 °C and 25 °C, the average longevity, parasitism rate, sex ratio, emergence duration, and offspring lifespan of adult *B*. *communis* were all superior compared to 15 °C and 35 °C. In addition, the parasitoids’ average longevity was considerably extended by 25 °C, and 60% RH. 25 °C could greatly increase the number of daily parasitized *Aphis gossypii*, while humidity, as well as the interaction between humidity and temperature, had no significant influence on parasite capacity. Consequently, our research indicated that 25 °C could improve the fitness of *B. communis*. These findings provide valuable insights for the indoor population expansion and field release of *B*. *communis*, and are crucial for strengthening aphid biological control strategies.

## 1. Introduction

*Aphis gossypii* Glover (Hemiptera: Aphididae) is a key pest of cotton in China, causing direct physical damage by extracting carbohydrates and amino acids from plant phloem, and facilitating the transmission of plant viral diseases, resulting in huge economic losses [1,2]. Currently, the prolonged and excessive use of neonicotinoid as well as pyrethroid insecticides not only induces resistance in aphids but also triggers their resurgence, posing a series of negative issues concerning human and animal safety, the ecological environment, and conservational biological control [3,4,5]. Therefore, there is an urgent need to develop more sustainable and effective control strategies for managing *A. gossypii*.

*Binodoxys communis* Gahan (Hymenoptera: Braconidae) is a dominant parasitoid of *A. gossypii* in Xinjiang Uygur Autonomous Region and northern China [1,6]. As an endoparasitoid, *B. communis* lays eggs within the host aphids, utilizing the host’s nutrients to support its own growth and development, which ultimately leads to the death of the host, effectively controlling and limiting aphid populations [7]. Previous studies have shown that when feeding on glucose, the parasitism rate of the parasitoid could reach 69.54 ± 3.34% [8]. In addition, when *A. gossypii* was parasitized by *B. communis*, its developmental duration was significantly prolonged, and fecundity was reduced [9]. Consequently, *B. communis* shows great potential as an environmentally friendly and sustainable biocontrol agent for preventing *A. gossypii* outbreaks.

In the context of the global environment, much of the focus has been on the effects of abiotic factors, such as temperature and humidity, on organisms [10]. Parasitoids, as ectotherms, are particularly influenced by temperature and humidity in terms of their survival, growth, and development, and the development of parasitic wasps is typically more sensitive to temperature than that of their hosts [11,12]. Temperature, in particular, has diverse effects on parasitoids, including impacts on longevity, fecundity, sex allocation, behavior, and morphology [13]. Previous studies have shown that the longevity of all five *Trichogramma* species, *Trichogramma pretiosum* Riley, *Trichogramma atopovirilia* Oatman and Platner, *Trichogramma acacioi* Brun, Moraes and Soares, *Trichogramma lasallei* Pinto, and *Trichogramma rojasi* Nagaraja and Nagarkatti, decreased with increasing temperature from 14 to 30 °C [14]. Increasing temperature significantly shortened the developmental time of the hymenopteran *Cotesia congregata* (Say) within the host *Manduca sexta* (L.), which was longer in the 20 °C rearing treatment than at 25 °C or 30 °C [15]. The parasitism rate of *Aphidius ervi* Haliday on *Aphis pomi* de Geer was significantly affected by temperature, ranging from a low of 10.8% at 15 °C to a high of 16.9% at 25 °C [16]. Exposure to extreme temperatures can even result in lethal or sublethal outcomes [13,17,18,19]. In addition, humidity levels play a crucial role in determining the availability of moisture that insects acquire from their environment [20]. Humidity tends to exhibit more variability than temperature, making it harder to show a clear trend in humidity than in temperature [20]. However, our knowledge of how humidity affects natural enemies of herbivorous hosts remains limited. Therefore, it is essential to investigate the effects of both temperature and humidity and their interaction on parasitoids.

We investigated the effects of temperature, as well as humidity, on the longevity, parasitism, progeny development, and functional responses of *B. communis*. The goal was to screen the temperature and humidity that are favorable for the development and reproduction of *B. communis*, providing a theoretical basis for selecting a suitable timing for indoor population expansion and field release of *B. communis*, so as to better apply to the biological control of aphids.

## 2. Materials and Methods

### 2.1. Biological Materials

The population of *A. gossypii* was collected in 2021 from cotton plants (CCRI49) at Korla Experimental Station, Chinese Academy of Agricultural Sciences (CAAS), Xinjiang Province, China. These aphids had been continuously reared for >1 year with cotton plants in plastic basins (20 × 10 × 6 cm) at 25 ± 1 °C, 60 ± 5% RH, and 16:8 h (L:D) photoperiod.

Mummified aphids were collected from a field at the Korla Experimental Station, CAAS, in the summer of 2021. Then, adults of *B. communis* were reared with a 10% honey solution for more than 10 generations, and cotton aphids *A. gossypii* were provided as the host every day at 25 ± 1 °C, 60 ± 5% RH and 16:8 h (L:D) photoperiod.

Cotton (CCRI49) seeds were obtained from the Institute of Cotton Research of CAAS and sown in a greenhouse at Korla Experimental Station, CAAS, under the following conditions: 26 ± 1 °C, 60 ± 10% RH, 16:8 h (L:D) photoperiod. Plants used for these tests were at the 2-true leaf growth stage.

### 2.2. Under Different Constant Temperature and Fluctuating Temperature

#### 2.2.1. Temperature Settings

To investigate the effect of temperature on the fitness of *B. communis*, we set different constant temperatures (CTs) and fluctuating temperatures (FTs): (1) CTs, including 15 °C, 20 °C, 25 °C, 30 °C, and 35 °C; (2) FT, 13–36 °C (starting from 13 °C at 20:00 every day and increasing by 1 °C per hour, with an average temperature of 24.5 °C). The relative humidity and photoperiod levels in the incubators were 60 ± 10% RH and 16:8 h (L:D), respectively.

#### 2.2.2. Average Longevity Under Different Temperatures

Two plexiglass rearing cages (30 × 30 × 25 cm) were placed in each incubator at different temperatures. 40 1 d-old female *B. communis* individuals, as well as 40 1 d-old males, were released in each cage and kept separately, fed with 10% honey solution. We checked parasitoid survival twice daily (at 8 a.m. and 8 p.m.) to record the number of dead wasps. Dead individuals were removed at each observation, and the honey solution was replaced. Each treatment was replicated five times, with 40 females and 40 males observed in each replicate until all wasps died. The average longevity of the parasitoid was then calculated.

#### 2.2.3. Fitness Assessment

Under different temperature conditions, 60 1st–2nd instar *A. gossypii* hosts were exposed to one 2 day-old mated female *B. communis* in 20 × 12 × 8 cm rectangular plastic boxes for life-history experiments. The parasitoids were supplied with a 10% honey solution, while the aphids were provided with fresh cotton leaves that were kept moist with agar. After 24 h, parasitoids were removed, while the hosts were kept and fed until death. The formation of mummies was observed every 24 h, and the mummies were carefully removed into 1.5 mL sealed tubes that were gauze-sealed and stored at their respective previous experimental temperatures. The total number of mummies was recorded to calculate the parasitism rate, the emergence time of the parasitoid wasps was recorded to calculate the emergence duration, the number of emerged parasitoid adults was recorded to calculate the emergence rate, the sex of the newly emerged wasps was determined, and the proportion of female wasps was calculated. The survival time of wasps after emergence was also recorded to calculate their average longevity of offspring. Four replications were set for each temperature, with 60 aphids used as hosts for each replicate.

### 2.3. Under Different Temperature and Relative Humidity Combinations

#### 2.3.1. Temperature and Humidity Settings

We set nine different temperatures and humidity combinations by adjusting the incubator: (1) 15 °C, 40%RH; (2) 15 °C, 60%RH; (3) 15 °C, 80%RH; (4) 25 °C, 40%RH; (5) 25 °C, 60%RH; (6) 25 °C, 80%RH; (7) 35 °C, 40%RH; (8) 35 °C, 60%RH; (9) 35 °C, 80%RH. The photoperiod in the incubators was 16:8 h (L:D), and a thermohygrometer (Xiaomi Co., Ltd., Beijing, China) was placed in each incubator to monitor temperature and humidity.

#### 2.3.2. Average Longevity Under Different Temperatures and Humidity

Two plexiglass rearing cages (30 × 30 × 25 cm) were placed in each incubator at different temperatures and humidity, and 40 1 d-old female *B. communis* individuals, as well as 40 1 d-old males, were released in each cage and kept separately, fed with 10% honey solution. We checked parasitoid survival twice daily (at 8 a.m. and 8 p.m.) to record the number of dead wasps. Dead individuals were removed at each observation, and the honey solution was replaced. Each treatment was replicated five times, with 40 females and 40 males observed in each replicate until all wasps died. The average longevity of the parasitoid was then calculated.

#### 2.3.3. Parasite Performance

In total, 60 1st–2nd instar *A. gossypii* hosts were placed in rectangular plastic boxes (20 × 12 × 8 cm) and exposed to a 2 day-old female *B. communis* for parasitism under various temperature and humidity conditions. The parasitoids were given a 10% honey solution, and the aphids were given fresh cotton leaves that had been moistened with agar. The parasitoids were eliminated after a day, but the hosts were retained until they passed away. The development of mummified aphids was monitored every 24 h, and those that had formed were taken out and noted. The number of mummified aphids was used to calculate the parasitism rate. When no mummified aphids formed for five days in a row, the experiment was over. Each treatment contained 60 host aphids and was carried out three times.

In total, 30, 50, 100, and 150 1st–2nd instar *A. gossypii* were offered as hosts for a 2 day-old female *B. communis* in order to evaluate the impact of temperature and humidity on the parasitoid wasps’ capacity for parasitism. The parasitoids were eliminated during a 24 h period of parasitism. The development of mummified aphids was monitored every 24 h, and those that were found were eliminated. The quantity of mummified aphids was noted, and their arrival was regarded as a successful parasitism occurrence. Three duplicates of each treatment were made.

### 2.4. Data Analysis

The Shapiro–Wilk test was conducted to assess the normality of the data before analysis. The percentages were transformed using the arcsine square root transformation to meet the requirements of variance analysis. The average longevity, parasitism rate (the percentage of produced mummies to surviving aphids), emergence rate (the percentage of parasitoid adults successfully emerging from mummies), and sex ratio (the percentage of female adults among offspring) under different temperature treatments followed a normal distribution, and were analyzed using one-way ANOVA, followed by Tukey’s HSD test. The emergence duration (the number of days from mummy to adult for the parasitoids), along with the longevity of offspring (the number of days parasitoid offspring lived from birth to death), did not conform to a normal distribution and were analyzed by the non-parametric Kruskal–Wallis test. The effects of temperature, humidity, and their interaction on average longevity and the average number of aphids parasitized per parasitoid per day were evaluated by two-way ANOVA.

In addition, the functional response of *B. communis* on *A. gossypii* was fitted using the Holling-II equation: *N_a_* = *aNT*/(1 + *aT_h_N*) [21]. Among them, *N_a_* represented the number of hosts, *N* was the host density, *T* = 1 h, *a* was the instantaneous attack rate, and *T_h_* was the time for wasps to handle each host.

Data were analyzed using SPSS 25.0 with a significance level of 0.05, and all figures were generated using Graphpad Prism 9.0.

## 3. Results

### 3.1. Under Different Temperatures

#### 3.1.1. Average Longevity Under Different Temperatures

**Figure 1:**
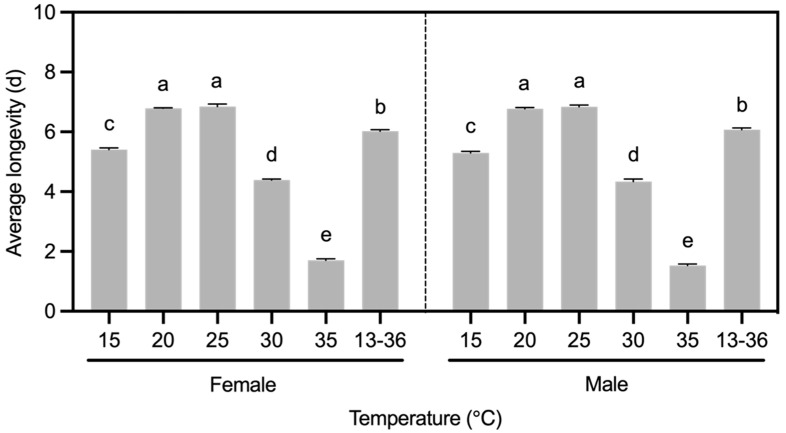
Effects of temperature on longevity of female and male adults of *B. communis*. Data in the table were Mean ± SE of five replicates, with 40 female wasps and 40 male wasps observed in each replicate. Values bearing different lowercase letters were significantly different (*p* < 0.05, Tukey’s HSD).

The average longevity of female (*F*_5,24_ = 1353.00, *p* < 0.001) and male (*F*_5,24_ = 1106.51, *p* < 0.001) *B. communis* was significantly affected by temperature, and both sexes of parasitoid wasps responded consistently to temperature in terms of longevity (Figure 1). Under constant temperature conditions, the average longevity of *B. communis* showed a trend of first extending and then shortening as the temperature increased. At 25 °C, the average longevity of *B. communis* reached its maximum (Female: 6.85 ± 0.08 d; Male: 6.84 ± 0.06 d). However, the average longevity of adult wasps was significantly shortened at 30 °C and 35 °C, especially at 35 °C where the longevity was the shortest, only 22.38~24.88% of that at 25 °C. At 15 °C, the average longevity of adults was shorter but still significantly longer than that at 30 °C and 35 °C. Under variable temperature conditions (13–36 °C), adults’ average longevity was second only to the maximum, significantly higher than that under low and high temperatures (Figure 1).

#### 3.1.2. Reproductive Capability

**Figure 2:**
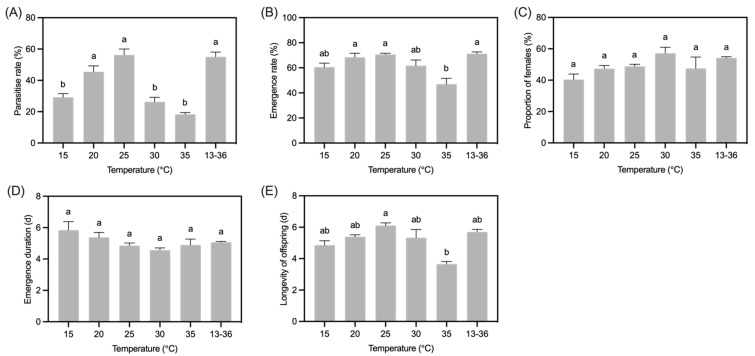
The effects of temperature on parasitism rate (**A**), emergence rate (**B**), proportion of females (**C**), emergence duration (**D**), and offspring longevity (**E**) of *B. communis*. Data in the table were Mean ± SE of four replicates, with 60 aphids as hosts in each replicate. The values bearing different lowercase letters were significantly different (*p* < 0.05, Tukey’s HSD).

Temperature significantly affected the parasitism rate of *B. communis* (*F*_5,18_ = 28.99, *p* < 0.001). At 15 °C, 20 °C, and 25 °C, the parasitism rate of *B. communis* increased with rising temperatures, reaching its maximum of 56.25 ± 7.62% at 25 °C. However, as the temperature continued to rise to 30 °C and 35 °C, the parasitism rate showed a decreasing trend, with the rate at 35 °C being significantly lower than that at other temperatures, only 18.33 ± 1.18%. Under variable temperature conditions, the parasitism rate was 55.00 ± 6.09%, second only to that at 25 °C (Figure 2A).

Temperature also had a significant effect on the emergence rate of *B. communis* (*F*_5,18_ = 7.49, *p* < 0.01). Under constant temperature conditions, the emergence rate was relatively high at 20 °C and 25 °C, with rates of 68.34 ± 3.28% and 70.65 ± 1.93%, respectively. As the temperature further increased, the emergence rate decreased, with the lowest rate at 35 °C, only 66.48% of that at 25 °C. The emergence rate was the highest under variable temperature conditions, reaching 71.24 ± 2.12%, showing no difference from that at 25 °C (Figure 2B).

In addition, different temperatures had no significant effect on the female proportion of *B. communis* (*F*_5,18_ = 2.37, *p* = 0.08). Under constant temperature conditions, the sex ratio showed a trend of first increasing and then decreasing with the increase in temperature. The sex ratio reached its maximum at 30 °C (57.13 ± 3.85%), and was the lowest at 15 °C (40.39 ± 7.11%). The sex ratio under variable temperature conditions was slightly lower than that at 30 °C (54.26 ± 0.70%; Figure 2C).

Temperature had no significant effect on the emergence duration of adult offspring of *B. communis* (*H* = 8.56, *p* = 0.13). At 15–30 °C, the emergence duration of adult offspring decreased with temperature increased, with the shortest emergence duration observed at 30 °C, averaging 4.57 days. However, the emergence duration was slightly prolonged at 35 °C (4.90 d), which was comparable to the duration at 35 °C (5.06 d; Figure 2D).

Offspring longevity varied significantly depending on temperature exposure (*H* = 18.25, *p* = 0.01). As the temperature rose from 15 °C to 25 °C, the longevity of the progeny increased, with the longest lifespan recorded at 25 °C (6.11 d). At 35 °C, the shortest progeny lifespan was 3.66 d. Offspring that were raised at 25 °C lived noticeably longer than those raised at 35 °C. Adult offspring of *B. communis* can survive for 5.70 ± 0.19 d in a temperature range of 13–36 °C (Figure 2E).

### 3.2. Under Different Temperatures and Humidity Conditions

#### 3.2.1. Average Longevity Under Different Temperatures and Humidity

The average longevity of *B. communis* was significantly influenced by temperature (two–way ANOVA, female: *F*_2,81_ = 32,482.94, *p* < 0.001; male: *F*_2,81_ = 26,653.90, *p* < 0.001), relative humidity (two–way ANOVA, female: *F*_2,81_ = 634.77, *p* < 0.001; male: *F*_2_^,^_81_ = 504.02, *p* < 0.001), and their interactions (two–way ANOVA, female: *F*_4,81_ = 36.91, *p* < 0.001; male: *F*_4,81_ = 124.47, *p* < 0.001). The average longevity of both female (40%RH: *F*_2,12_ = 3619.69, *p* < 0.001; 60%RH: *F*_2,12_ = 6163.42, *p* < 0.001; 80%RH: *F*_2,12_ = 1873.40, *p* < 0.001) and male (40%RH: *F*_2,12_ = 4676.61, *p* < 0.001; 60%RH: *F*_2,12_ = 3861.97, *p* < 0.001; 80%RH: *F*_2,12_ = 1611.65, *p* < 0.001) wasps at 25 °C was significantly longer than at 15 °C and 35 °C. At 60% RH, the average longevity of both female (15 °C: *F*_2,12_ = 87.27, *p* < 0.001; 25 °C: *F*_2,12_ = 116.69, *p* < 0.001; 35 °C: *F*_2,12_ = 107.43, *p* < 0.001) and male (15 °C: *F*_2,12_ = 100.36, *p* < 0.001; 25 °C: *F*_2,12_ = 143.60, *p* < 0.001; 35 °C: *F*_2,12_ = 17.12, *p* < 0.001) wasps was significantly greater than at 40% RH and 80% RH. In summary, under conditions of 25 °C and 60% RH, female and male wasps had the longest average longevity, reaching 6.88 ± 0.07 d and 6.96 ± 0.10 d, respectively (Table 1).

#### 3.2.2. Parasitism Ability

Temperature had a significant impact on the daily parasitism rate of *B. communis*. At host densities of 30 (40%RH: *F*_2,6_ = 27.46, *p* = 0.001; 60%RH: *F*_2,6_ = 12.53, *p* = 0.007; 80%RH: *F*_2,6_ = 25.16, *p* = 0.001), 50 (40%RH: *F*_2,6_ = 9.59, *p* = 0.014; 60%RH: *F*_2,6_ = 9.21, *p* = 0.015; 80%RH: *F*_2,6_ = 10.99, *p* = 0.010), 100 (40%RH: *F*_2,6_ = 32.72, *p* = 0.001; 60%RH: *F*_2,6_ = 27.66, *p* = 0.001; 80%RH: *F*_2,6_ = 21.43, *p* = 0.002), and 150 (40%RH: *F*_2,6_ = 40.51, *p* < 0.001; 60%RH: *F*_2,6_ = 34.38, *p* = 0.001; 80%RH: *F*_2,6_ = 26.00, *p* = 0.001) individuals per box, the daily average number of parasitized aphids at 25 °C was significantly higher than the daily average parasitism rate under low and high-temperature conditions. Humidity (*p* > 0.05) and the interaction between humidity and temperature (*p* > 0.05) had no effect on mating duration (Table 2).

**Figure 3:**
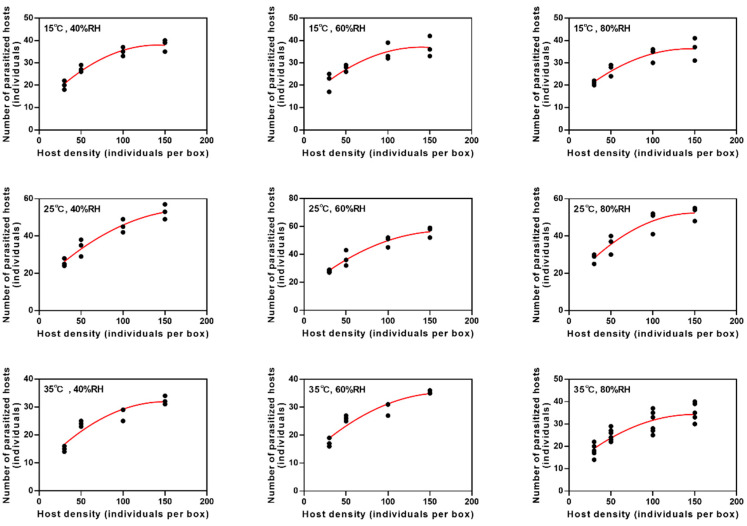
The fitting curve of parasitic functional response of *B. communis* under different temperatures and relative humidity. The solid red line in the graph represents the Holling-II model curve. The data points represent the actual amount of parasitism of *B. communis* under the host density.

The best-fitting curve of the average daily parasitism rate of *B. communis* under different temperatures and humidity conformed to the Holling-II equation. Also, the number of parasitized aphids increased with the increasing aphid density at different temperatures and humidity and then gradually stabilized (Figure 3).

According to the functional response parameters at various temperatures and relative humidity, the shortest time for *B. communis* to parasitize one cotton aphid was at 35 °C and 80% RH (*T_h_* = 0.0131, about 0.3144 h), while the longest was at 15 °C and 80% RH (*T_h_* = 0.0222, about 0.5328 h). At 25 °C and 80% RH, the instantaneous attack rate was at its highest (1.5889), whereas at 35 °C and 80% RH, it was at its lowest (0.3085). At 25 °C, the maximum daily parasitism was 1.33~1.80 times that at 20 °C and 35 °C, reaching its peak (69.97) at 25 °C and 80% RH (Table 3).

## 4. Discussion

In this study, we investigated how the life–history traits of *B. communis* were affected by different constant and fluctuating temperatures, as well as different combinations of temperature and humidity. We showed that temperature and humidity strongly affected the fitness of *B. communis*.

Ambient temperature is the source of heat energy in insects and an important factor affecting their growth, development, reproduction, and other activities [22]. The development of *B. communis* requires moderate temperatures, both high and low temperatures, to prevent its normal growth and development. Our results showed that the longevity of *B. communis* reached its maximum at 20 °C and 25 °C, whereas at 30 °C and 35 °C, adult wasps had a significantly shorter lifespan, with the shortest longevity observed at 35 °C, where it was only 22.38~24.88% of that at 25 °C. Similarly, at 20 °C and 25 °C, parasitoid wasps outperformed 15 °C and 35 °C in terms of parasitism rate, female ratio, and longevity of offspring. Our experimental results were consistent with many previous studies. For example, the parasitism rates of third instar *Loxostege sticticalis* L. by *Zele chlorophthalmus* (Spinola) at 21 °C and 25 °C were significantly higher than those at 17 °C and 29 °C, while there was no parasitism at 33 °C [23]. When cotton aphids on cucumber (*Cucumis sativus* L.) seedlings were used as hosts, the sex ratio of *B. communis* was higher in males at low (17.5–22.5 °C) or high (32.5 °C) temperatures, and females at 25–30 °C [24]. In addition, progeny production tended to be reduced at 14 °C and 30 °C for *T. pretiosum*, *T. atopovirilia*, *T. acacioi*, *T. lasallei*, and *T. rojasi* than at 18 °C, 21 °C, and 26 °C [14]. Extreme high temperatures (EHTs) can cause thermal injury, protein denaturation, and depress the diversity and activity of symbiotic bacteria, leading to a series of disorders at the molecular, biochemical, and physiological levels in insects [19]. In addition, cold exposure, either lethal or sublethal, is a major factor shaping several life–history traits in parasitoid insects, such as longevity, fecundity, sex allocation, and behavioral consequences [13,25,26].

Temperature is one of the key elements influencing the growth and development of insects. Previous research showed that insect development is often proportional to temperature within a specific range; going above or below this range will slow or even stop insect development [27,28]. The development time of parasitoid *Venturia canescuns* Gravenhorst larvae (from parasitism to pupation), pupae (from pupation to adult emergence), and the total development time from parasitism to emergence significantly decreased with increasing temperature in the temperature range of 17.5~27.5 °C [29].When parasitizing cucumber aphids, the developmental period of *B. communis* eggs and mummies considerably shortened with temperatures rising from 17.5 °C to 32.5 °C, and development stopped at the high temperature of 35 °C [24]. This phenomenon occurs not only in parasitic wasps but also in predatory species such as *Cheilomenes sexmaculata* (F.) and *Oenopia conglobata* (L.) [30,31]. Our results indicated that the emergence time of *B. communis* decreased with increasing temperature within 15–30 °C. At 35 °C, the emergence time of the parasitoid was longer than at 30 °C. Although our results showed the same trend as previous studies, temperature had no significant effect on the emergence duration of adult offspring. Previous results showed that *Trichogramma chilonis* Ishii and *Trichogramma evanescens* Westwood completed development at 13 °C, 18 °C, 25 °C and 34 °C, but *Trichogramma bournieri* Pintureau and Babault and *Trichogramma* sp. nr. *mwanzai* Schulten and Feijen failed to complete development at the lowest temperature (13 °C) [32]. In addition, the preoviposition period of *Tetranychus truncatus* Ehara was not affected by temperature [33]. These variations could be explained by the different ways that different individuals and developmental stages react to temperature.

Compared to CTs, FTs may provide a more accurate assessment of the developmental biology of insects [34]. We investigated the effects of different CTs and FTs on the growth and development of *B. communis*, and our results showed that the fitness of parasitoids under FTs was second only to that observed under optimal CTs. This may be due to direct cold or heat damage caused by FTs with stressful temperature fluctuations, as well as the subsequent physiological and biochemical costs of repair [35,36].

The effect of nine combinations of temperature and humidity on the longevity of the parasitoid wasps showed that the highest longevity of the parasitoid was observed at 25 °C and 60%RH, with females and males living for 6.88 and 6.96 d, respectively. Both low humidity (RH = 40%) and high humidity (RH = 80%) conditions had a negative effect on the survival and parasitism of *B. communis* individuals, which was detrimental to the occurrence of their populations. Humidity may affect the lifespan of insects by altering their energy state and causing molecular damage. In addition, changes in humidity could indirectly affect the natural enemies through changes in host plant metabolism and physiology [37].

Functional response is one of the most important indicators for evaluating the ability of parasitoid wasps to control their hosts [38]. The rational use of functional response modeling of parasitic natural enemies can significantly improve the efficiency of pest control by parasitic natural enemies in the field. In this experiment, multiple host densities were set under nine temperature and humidity combinations, and it was found that the parasitism of *B. communis* on *A. gossypii* conformed to the functional Holling-II model. The fitting curves showed that the wasps had strong parasitism ability on low instar aphids under different temperatures and humidity, and its parasitic number increased with the increase in host density and gradually stabilized. The results of this study were useful for predicting the potential of parasitic wasps to control cotton aphids based on changes in field temperatures. However, since the experiment was conducted indoors using cotton leaves in plastic bins, caution needs to be exercised when replicating it in the field as the environment is more complex.

## 5. Conclusions

From an ecological perspective, this study found that 25 °C could improve the fitness of *B. communis*, offering valuable insights for indoor population expansion and field release of *B. communis*. Further field experiments are necessary to elucidate the effects of different temperatures and humidity conditions on the biological control of *B. communis*.

## Figures and Tables

**Table 1:** The effects of temperature and humidity on average longevity of female and male adults of *B. communis*.

Temperature (°C)	Relative Humidity (%)	Average Longevity (d)	
		Female	Male
15	40	5.05 ± 0.12 bB	5.02 ± 0.05 bB
	60	5.45 ± 0.07 aB	5.23 ± 0.09 aB
	80	4.48 ± 0.12 cB	4.39 ± 0.11 cB
25	40	6.69 ± 0.07 bA	6.79 ± 0.10 bA
	60	6.88 ± 0.07 aA	6.96 ± 0.10 aA
	80	5.92 ± 0.13 cA	5.70 ± 0.14 cA
35	40	1.79 ± 0.03 bC	1.78 ± 0.07 bC
	60	1.94 ± 0.06 aC	1.70 ± 0.06 aC
	80	1.44 ± 0.06 cC	1.54 ± 0.05 cC
Temperature		**	**
Relative humidity		**	**
Temperature × Relative humidity		**	**

Note: Data in the table were Mean ± SE of three replicates. Different lowercase letters indicate significant differences in the number of cotton aphids parasitized daily by *B. communis* under different relative humidity; different uppercase letters indicate significant differences in the number of hosts parasitized per day under different temperatures (*p* < 0.05, Tukey’s HSD). “**” indicates a highly significant difference as determined by two–way ANOVA analysis (*p* < 0.01).

**Table 2:** The number of cotton aphids parasitized daily by *B. communis* under different temperatures and relative humidity conditions.

Temperature (°C)	Relative Humidity (%)	Number of Host (Individuals per Box)			
		30	50	100	150
15	40	20.00 ± 1.15 aB	27.33 ± 0.88 aAB	35.00 ± 1.15 aB	38.00 ± 1.53 aB
	60	21.67 ± 2.40 aB	27.67 ± 0.58 aAB	34.67 ± 2.19 aB	37.00 ± 2.65 aB
	80	21.00 ± 0.58 aB	27.00 ± 1.53 aB	33.67 ± 1.86 aB	36.33 ± 2.91 aB
25	40	25.67 ± 1.21 aA	34.00 ± 2.65 aA	45.33 ± 2.03 aA	53.00 ± 2.31 aA
	60	26.33 ± 1.21 aA	32.33 ± 2.03 aA	46.00 ± 2.65 aA	53.33 ± 2.96 aA
	80	28.00 ± 1.53 aA	35.67 ± 2.96 aA	48.00 ± 3.51 aA	52.33 ± 2.19 aA
35	40	15.00 ± 0.58 aC	24.00 ± 0.58 aB	27.67 ± 1.33 aC	32.33 ± 0.88 aB
	60	16.67 ± 1.20 aC	25.67 ± 0.88 aB	29.00 ± 1.15 aB	34.00 ± 1.53 aB
	80	16.33 ± 1.20 aC	23.00 ± 0.57 aB	26.67 ± 0.88 aB	31.00 ± 1.00 aB
Temperature		**	**	**	**
Relative humidity		ns	ns	ns	ns
Temperature × Relative humidity		ns	ns	ns	ns

Note: Data in the table were Mean ± SE of three replicates. Different lowercase letters indicate significant differences in the number of cotton aphids parasitized daily by *B. communis* under different relative humidity; different uppercase letters indicated significant differences in the number of hosts parasitized per day under different temperatures (*p* < 0.05, Tukey’s HSD). “**” indicates a highly significant difference as determined by two–way ANOVA analysis (*p* < 0.01), and “ns” indicates no difference (*p >* 0.05).

**Table 3:** Parasitic functional response parameters of *B. communis* under different temperatures and relative humidity.

Temperature/Relative Humidity	Disk Equation of Functional Response	Correlation Coefficient	Attack Rate (*a*)	Handling Time (*T_h_*)	Parasitical Efficiency	Daily Maximum Parasitism (*N_a_* max)
15 °C/40%	*N*_a_ = 1.1219 *N*_0_/(1 + 0.0222 *N*_0_)	0.8913	1.1219	0.0198	56.66	50.54
15 °C/60%	*N*_a_ = 1.3800 *N*_0_/(1+ 0.0302 *N*_0_)	0.7246	1.3800	0.0219	63.01	45.70
15 °C/80%	*N*_a_ = 1.3223 *N*_0_/(1+ 0.0295 *N*_0_)	0.7563	1.3223	0.0222	60.38	44.82
25 °C/40%	*N*_a_ = 1.3435 *N*_0_/(1+ 0.0192 *N*_0_)	0.7443	1.3435	0.0143	93.95	69.97
25 °C/60%	*N*_a_ = 1.3834 *N*_0_/(1+ 0.0204 *N*_0_)	0.7228	1.3834	0.0147	94.11	67.81
25 °C/80%	*N*_a_ =1.5889 *N*_0_/(1+ 0.0237 *N*_0_)	0.6294	1.5889	0.0149	106.64	67.04
35 °C/40%	*N*_a_ = 0.7778 *N*_0_/(1+ 0.0166 *N*_0_)	1.2857	0.7776	0.0214	36.34	46.86
35 °C/60%	*N*_a_ = 0.9162 *N*_0_/(1+ 0.0198 *N*_0_)	1.0925	0.9162	0.0216	42.42	46.27
35 °C/80%	*N*_a_ = 0.3085 *N*_0_/(1+ 0.0079 *N*_0_)	3.2416	0.3085	0.0131	25.55	39.05

## Data Availability

All data analyzed in this study are included in this article.
